# Risk Factors of Open Surgery Conversion in Laparoscopic Partial Nephrectomy to Achieve Nephron Sparing

**DOI:** 10.1245/s10434-024-15106-1

**Published:** 2024-03-08

**Authors:** Emin Taha Keskin, Osman Can, Harun Özdemir, Merve Şam Özdemir, Özgür Deniz Tataroğlu, Abdülmuttalip Şimşek

**Affiliations:** 1 Department of Urology, Basaksehir Cam and Sakura City Hospital, Istanbul, Turkey; 2 Department of Radiology, Basaksehir Cam and Sakura City Hospital, Istanbul, Turkey

**Keywords:** Conversion to open surgery, Laparoscopy, Partial nephrectomy, Renal cell carcinoma

## Abstract

**Objectives:**

We aimed to evaluate the risk factors for the conversion from laparoscopic partial nephrectomy (LPN) to open surgery to achieve partial nephrectomy (PN).

**Methods:**

Data from patients who underwent LPN between June 2020 and September 2023 were analyzed retrospectively. Patients in whom the PN procedure could be completed laparoscopically were recorded as the ‘Fully Laparoscopic’ (FL) group (*n* = 97), and those converted to open surgery from laparoscopy were recorded as the ‘Conversion to Open’ (CTO) group (*n* = 10). The demographic and pathologic variables were compared between groups. Regression analyses were used to define predictor factors, and receiver operating characteristic analysis was used to define the cut-off value of the surgical bleeding volume.

**Results:**

Conversion to open surgery was found in 10/107 patients (9.3%). There was no statistical difference between groups in demographic and pathologic variables. Intraoperative blood loss volume, upper pole localized tumor, and posterior localized tumor were found to be statistically higher in the CTO group (*p* = 0.001, *p =* 0.001, and *p =* 0.043, respectively). Furthermore, these factors were only found to be statistically significant predictors of conversion to open surgery in both univariate and multivariate regression analyses. 235 cc was found to be the cut-off value of intraoperative blood loss volume for predicting conversion to open surgery (*p =* 0.001).

**Conclusion:**

Using these predictive factors in clinical practice, treatment planning will lead to the possibility of starting the treatment directly with open surgery instead of minimally invasive options, and it may also provide a chance of being prepared for the possibility of conversion to open surgery peroperatively.

Renal cell carcinoma (RCC), the most commonly diagnosed solid lesion within the kidney, is found in around 3% of all cancers.^1^ Recently, a higher prevalence of small renal masses (SRMs) has been detected in patients for whom abdominal imaging is performed more frequently.^2^

The most preferred treatments for kidney tumors are surgical procedures. Partial nephrectomy (PN), a subtype of nephrectomy, is a technique in which only the tumor is removed with a negative surgical margin to preserve tumor-free renal parenchyma.^3^ Without compromising oncologic efficacy, laparoscopic PN (LPN) is gaining popularity as a minimally invasive alternative to open PN (OPN) for SRMs, with decreased morbidity.^4^

In order to achieve successful kidney sparing, the surgeon might need to switch from laparoscopy to an open procedure in a variety of conditions during LPN. During minimally invasive renal surgery, unplanned intraoperative conversions to open surgery could also have an effect on surgical planning, preoperative patient counseling, and cost-benefit analyses of laparoscopic surgery.

In the current literature, only a few studies have analyzed the risk factor of conversion to an open approach from LPN. Thus, with this study, we aimed to evaluate the risk factors of conversion from LPN to open surgery to achieve PN.

## Materials and Methods

**Fig. 1:**
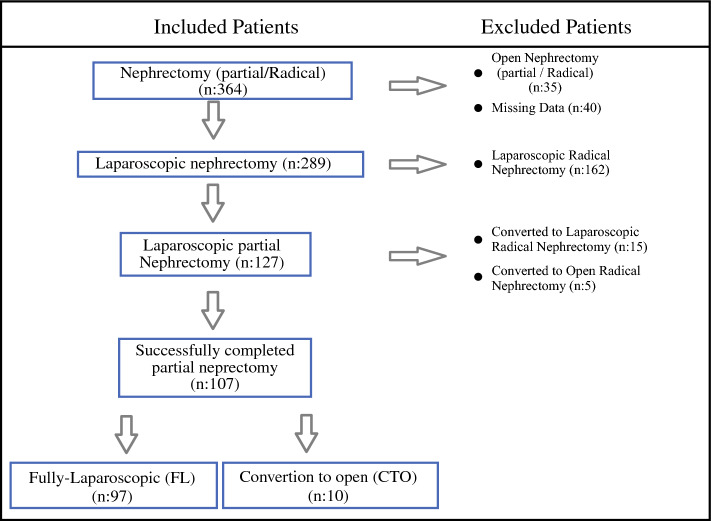
Study selection process

In this retrospective study, we enrolled data for patients who underwent LPN in our high-volume, top-level referral center between June 2020 and September 2023, after Institutional Review Board approval (KAEK/2023.09.424) was obtained. Only patients who underwent transperitoneal LPN for kidney tumors were included in our study. As defined in the flowchart shown in Fig. 1, patients who underwent other surgical procedures (open, robot-assisted, retroperitoneal), patients who were converted from LPN to radical nephrectomy (RN; open or laparoscopic) peroperatively for any reason, and patients with missing data were excluded from the study.

A total of 107 patients were enrolled in the study using the inclusion and exclusion criteria. According to the ability to convert to open surgery from laparoscopy, patients were divided into two different groups: patients whose PN procedure could be completed laparoscopically were recorded as the ‘Fully Laparoscopic’ (FL) group (*n* = 97), and patients who underwent PN by converting to open surgery because the PN procedure could not be completed laparoscopically were recorded as the ‘Conversion to Open’ (CTO) group (*n* = 10).

Patient demographic data, preoperative laboratory results and radiological findings, as well as pathological results were analyzed for both groups. Intraoperative blood loss was only recorded during laparoscopic surgery, as peroperative data. The amount of bleeding after conversion to open surgery was not calculated and was hence not included for analysis in this study.

During the laparascopic PN procedure, only the resection technique with warm ischemia was performed on all patients by experienced urologists with at least 5 years of surgical experience. All radiological images were evaluated by the same experienced uroradiologist, and all the pathological specimens were evaluated by the same experienced uropathologist.

### Statistical Analysis

Distribution of the patient data was tested for normality using the Kolmogorov–Smirnov test. Data are expressed as mean ± standard deviation for data with a normal distribution, or median [interquartile range] for non-normally distributed data. To compare groups, the *t*-test and Mann–Whitney *U* were used, and the Chi-square test was used to compare categorical variables. Univariate and multivariate regression analyses were used to define predictor factors of conversion from laparoscopic to open surgery. The cut-off value of the surgical bleeding volume, which can be used to predict conversion to open surgery peroperatively, was calculated by receiver operating characteristic (ROC) analysis. The specificity, sensitivity, and negative and positive predictive values (PPVs) were calculated for the cut-off value. In this study, data obtained from personal information forms and scales were transferred to a computer using the SPSS (Statistical Package Programme for Social Sciences) version 22.0 program (IBM Corporation, Armonk, NY, USA), and the data were analyzed using this program. A *p*-value was accepted as <0.05 at a 95% confidence interval.

## Results

The demographic and pathologic comparison results of all patients are described in Table 1. Conversion to open surgery from laparoscopic surgery was found in 10/107 patients (9.3%): two patients (20%) in whom laparoscopic dissection could not be performed due to adhesion; one patient (10%) due to renal vascular injury; one patient (10%) due to suspicion of positive surgical margins; one patient (10%) in whom the collecting system adjacent to the tumor was severely injured; and the remaining five patients (50%) were converted to open surgery due to bleeding from the tumor bed that could not be controlled laparoscopically for any reason.

**Table 1:** Demographic data, tumor features, peroperative data and pathologic results of all patients, and comparison of patient data between the fully laparoscopic (FL) and conversion to open (CTO) surgery groups

	Fully laparoscopic group [*n* = 97]	Conversion to open group [*n* = 10]	*p*-Value
Age, years (mean ± SD)	56.7 ± 11	53.6 ± 8.4	0.386*
Sex [*n* (%)]			
Male	59.8 (58)	90 (9)	0.060***
Female	40.2 (39)	10 (1)	
BMI, kg/m^2^ (mean ± SD)	27.3 ± 3.7	29.4 ± 3.1	0.086*
Preoperative laboratory results (mean ± SD)			
Creatinine, mg/dL	1.0 ± 0.8	0.9 ± 0.2	0.559**
eGFR	88.6 ± 21.9	88.8 ± 20	0.949**
Hemoglobin, mg/dL	13.4 ± 1.8	14.6 ± 1.7	0.055*
Platelet count, 10^3^/μL	274.7 ± 81.4	269.6 ± 88.9	0.852*
Comorbidities [*n* (%)]			
Diabetes mellitus	48.5 (47)	50 (5)	0.926***
Hypertension	49.5 (48)	50 (5)	0.975***
Cardiovascular diseases	34 (33)	40 (4)	0.891***
Cerebrovascular disease	5.2 (5)	0 (0)	NA
Lung diseases	15.5 (15)	0 (0)	NA
Anticoagulant use	15.5 (15)	10 (1)	0.645***
Antiplatelet use	33 (32)	30 (3)	0.848***
ASA score (mean ± SD)	2.7 ± 0.5	2.7 ± 0.4	0.859**
Previous abdominal surgery [*n* (%)]	5.2 (5)	10 (1)	0.526***
Renal vascular anomaly [*n* (%)]	5.2 (5)	20 (2)	0.071***
Tumor size, mm (mean ± SD)	34.3 ± 17	37.9 ± 17.5	0.618**
Tumor size [*n* (%)]			
<40 mm	75.3 (73)	60 (6)	0.296***
40 mm<	24.7 (24)	40 (4)	
Localization [*n* (%)]			
Upper pole	17.5 (17)	70 (7)	0.001***
Middle-lower pole	82.5 (80)	30 (30)	
Anterior	62.9 (61)	30 (3)	0.043***
Posterior	37.1 (36)	70 (7)	
Lateralite [*n* (%)]			
Right	54.6 (53)	60 (6)	0.686***
Left	45.4 (44)	40 (4)	
T stage [*n* (%)]			
T1a	75.3 (73)	60 (6)	0.422***
T1b	21.6 (21)	30 (3)	
T2a and above	3.1 (3)	1 (10)	
N stage			
N0	97 (94)	100 (10)	NA
N1	3 (3)	0 (0)	
Pathological results [*n* (%)]			
Clear cell RCC	68 (66)	80 (8)	0.726***
Papillary RCC	11.3 (11)	0 (0)	
Chromophobe RCC	6.2 (6)	10 (1)	
Angiomyolipoma	4.1 (4)	0 (0)	
Oncocytoma	5.2 (5)	0 (0)	
Other	5.2 (5)	10 (1)	
PSM *n* (%)]	10.3 (10)	0 (0)	NA
Nephrometry scores (mean ± SD)			
RENAL	6.25 ± 1.7	6.6 ± 1.8	0.532**
PADUA	7.13 ± 1.2	7.5 ± 2.3	0.124**
C-index	2.90 ± 1.3	2.65 ± 1.4	0.270**
Intraoperative blood loss volume, mL (mean ± SD)	165.1 ± 57.4	381 ± 208.3	0.001**
Warm ischemia time, min (mean ± SD)	23.9 ± 5.4	24.4 ± 4.6	0.594**

Bold indicates statistically significant (*p* < 0.05)

*ASA* American Society of Anesthesiologists, *BMI* body mass index, *eGFR* estimated glomerular filtration rate, *NA* not applicable, *PSM* positive surgical magrin, *RCC* renal cell cancer, *SD* standard deviation

*t-Test, **Mann–Whitney *U* test, ***Chi-square test

There was no statistical difference between groups in terms of age, sex, body mass index (BMI), American Society of Anesthesiologists (ASA) scores, presence of comorbidities, use of anticoagulants and/or antiplatelets, and history of previous abdominal surgery. Tumor size, lateralite (right or left kidney), pathological findings, nephrometry scores (PADUA, RENAL, and C-index), renal vascular anomaly, and warm ischemia time were found to be similar between groups (Table 1).

The upper pole localized tumor was found to be statistically higher than the mid-lower pole in the CTO group (*p =* 0.001). Similarly, the posterior localized tumor was found to be statistically higher than the anterior in the CTO group (*p =* 0.043) (Table 1).

Intraoperative warm ischemia time and volume of blood loss were compared between groups. There was no statistical difference between groups in warm ischemia time (*p* = 0.594), therefore intraoperative blood loss volume was found to be statistically higher in the CTO group (*p* = 0.001) (Table 1).

**Fig. 2:**
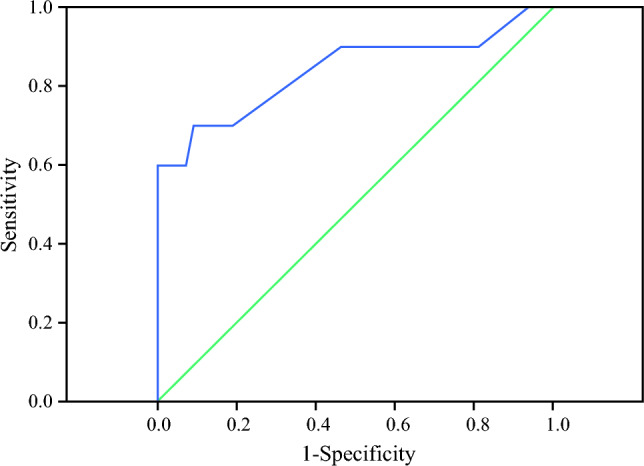
Receiver operating characteristic curve of intraoperative blood loss volume (mL) for predicting conversion to open surgery (AUC: 838)

To define an ideal cut-off value for intraoperative blood loss volume, 235 cc was found to be the cut-off value for predicting conversion to open surgery using ROC analysis (AUC 0.838; *p =* 0.001) (Fig. 2). Sensitivity, specificity, PPV, and negative predictive value (NPV) were calculated for this value as 70%, 90%, 87.5%, and 75%, respectively.

Regression analyses were used to define predictor factors of conversion from laparoscopic surgery to open surgery. Intraoperative blood loss volume, upper pole and posterior localized tumor were found to be statistically significant predictors of conversion to open surgery according to both univariate (*p* = 0.001, *p* = 0.008, and *p* = 0.028, respectively) and multivariate regression analysis (*p* = 0.001, *p* = 0.006, and *p* = 0.046, respectively) (Table 2).

**Table 2:** Univariate and multivariate regression models to predict conversion to open surgery from laparoscopy

	Univariate model			Multivariate model		
	OR	95% CI	*p*-Value	OR	95% CI	*p*-Value
		Lower–upper			Lower–upper	
Age, years (< 50, 50<)	1.92	0.500–7.365	0.055			
BMI, kg/m^2^ (<25, 25<)	1.13	1.050–1.231	0.596			
ASA score (1 and 2/3 and 4)	1.17	0.281–4.867	0.331			
Tumor size, mm (<40/40<)	0.49	0.128–1.896	0.883			
Lateralization (Left/right)	1.24	0.330–4.693	0.769			
Localization						
Anterior/posterior	0.25	0.062–1.040	**0.028**	0.75	0.007–0.843	**0.046**
Upper pole/mid and lower pole	10.9	2.575–46.82	**0.008**	12.5	2.062–76.74	**0.006**
Warm ischemia time, min (<25/25<)	0.42	0.114–1.622	0.284			
Intraoperative blood loss, mL (<235/235<)	2.29	1.114–2.653	**0.001**	5.49	5.217–57.81	**0.001**
Previous abdominal surgery	0.48	0.051–4.657	0.643			
Renal vascular anomaly	0.21	0.036–1.305	0.082			
Anticoagulant use	1.64	0.194–13.96	0.815			
Antiplatelet use	1.14	0.287–4.739	0.745			
Nephrometry scores						
RENAL (≤6/6<)	0.70	0.191–2.585	0.068			
PADUA (≤7/7<)	0.29	0.078–1.136	0.073			
C-index (≤2.5/2.5<)	2.38	0.582–9.775	0.768			

Bold indicates statistically significant (*p* < 0.05)

*ASA* American Society of Anesthesiologists score, *BMI* body mass index, *CI* confidence interval, *OR* odds ratio

## Discussion

Preserving healthy nephrons is well known and is related to a reduced risk of chronic kidney disease (CKD) and cardiovascular events.^5^ PN is a technique in which only the tumor is removed with a negative surgical margin to preserve tumor-free nephrons.^3^ In this way, numerous retrospective analyses have suggested using PN to lower cardiovascular-specific mortality and improve OS.^6^ Particularly for patients with pre-existing CKD, PN is the optimal treatment to reduce the risk of developing hemodialysis-dependent end-stage renal disease (ESRD).^7^ Decreasing the risk of developing these serious diseases is a well-known benefit of a PN rather than an RN. Achieving PN rather than RN as a treatment decision in particularly frail patients (such as bilateral renal tumors or patients with unilateral tumor with a poorly functioning contralateral kidney or absent of contralateral kidney) could sometimes be important to prevent CKD, which may develop or worsen postoperatively, despite the increased risk of perioperative complications.

LPN, which was first defined in 1993, is gaining popularity as a minimally invasive alternative to OPN without compromising oncologic efficacy.^4,8^ Although its use in clinics is increasing, not all PN cases could still be completed laparoscopically. By abandoning the LPN procedure, it is feasible to convert it to a laparoscopic RN (LRN) or converting to open surgery, which could be necessary for a wide range of reasons, including severe adhesion, bleeding, and vascular injury.^9^

The clinical impact of unplanned transformation has been demonstrated in several different situations. Unplanned intraoperative conversions could have an effect on surgical planning and preoperative patient counseling. Conversions were also found to be associated with higher morbidity and a prolonged recovery for patients. Particularly in the postoperative period, the length of the hospital stay was found to be longer in patients who experienced conversion to open surgery than in patients undergoing successful minimally invasive surgery (MIS). Similarly, 30-day mortality was found to be significantly higher in patients undergoing unplanned conversion.^10^As expected, it might also lead to additional costs for the healthcare system due to its various effects on clinics. We believe that the negative effects on the patient and the healthcare system can be minimized by predicting unplanned intraoperative conversions preoperatively for patients who require PN. With this study, we aimed to define the predictive risk factors of open conversion in LPN to achieve nephron-sparing to protect from possible complications of RN. To the best of our knowledge, this is the first study to determine predictive factors for conversion from LPN to OPN in order to achieve successful nephron-sparing.

LPN cases have the potential to require conversion to open surgery due to the increased technical complexity compared with RN cases. Regarding 79 patients undergoing LPN in 2003, Kim et al. reported one conversion to LRN and one conversion to OPN due to suspicion of positive margins and bleeding, respectively.^11^ Ramani et al. recorded the following in 2005: one LPN to LRN conversion due to persistent parenchymal hemorrhage, one LPN to OPN conversion due to tissue adhesions, and one LPN to ORN conversion resulting from bleeding in the presence of a normal contralateral kidney.^12^ One conversion from LPN to ORN was reported by Yoshikawa et al. in their study of 17 patients.^13^ In our study, conversion to open surgery from LPN was found in 9.3% of patients. Similar to the previous studies, the majority of patients (50%) were converted to open surgery due to bleeding from the tumor bed that could not be controlled laparoscopically. Excessive adhesion (20%), renal vascular injury (10%), suspicion of positive surgical margins (10%), and severely injured the collecting system (10%) were found to be reasons for conversion in our study.

Very few studies are currently available on this issue. Rais-Bahrami et al. showed that 15.6% of patients converted from LPN to LRN or open surgery. According to that study, the majority of patients who converted to open surgery underwent RN (14%) [LRN or ORN] and only 1.1% of patients were able to undergo OPN.^9^ In another study, Khanna et al. showed that the rate of unplanned conversions from LPN to open surgery was 6.4%.^10^ Despite the lack of data in the current literature, our study seems to have a higher conversion rate from LPN to OPN compared with other studies. It is thought that this high rate is due to serious comorbidities and more complex tumors (i.e., those located at the upper pole, posteriorly, or in close proximity to renal vascular systems) in patients who require PN rather than RN to be referred to our hospital, which is the highest level of reference hospital in our country.

A tendency for bleeding to occur more frequently in elderly patients was typically believed to be the result of platelet or coagulation factor function abnormalities. As a result, persistent bleeding from the tumor bed after the clamps were removed from the hilum was seen to be more common in elderly patients. Because of this high tendency to bleed, it was found that LPN in older patients (70 years of age) was significantly more likely to require conversion to LRN.^9^ Contrary to the literature, a patient’s age, prevalence of renal vascular anomalies, and use of anticoagulants and/or antiplatelets were not statistically significant differences between groups in our study. However, in our study, it was shown that in 50% of patients who converted to open surgery, this was as a result of intraoperative bleeding. High intraoperative blood loss was detected at a higher rate in patients who converted to open surgery. Using the ROC analysis, we found that 235 cc was the cut-off value for predicting conversion to open surgery. In clinical practice, it should be taken into consideration that since the amount of intraoperative bleeding is not a parameter that can be predicted in preoperative patient evaluation, it cannot be used in preoperative planning; however, this value of 235 cc may be a guide for the surgeon in making the decision to convert to open surgery peroperatively.

The relationship between the pathological features of the tumor and conversion to open surgery has been evaluated in a limited number of studies. Higher clinical *T* stage (*T*3–*T*4), clinical *N*1 disease, and larger tumor size were found to be associated with unplanned open conversion from both LPN and LRN.^10^ In our study, patients who converted from LPN to OPN in order to achieve the PN were compared; unlike other studies, no significant statistical relationship was found between tumor *T* stage, *N* stage, tumor size, nephrometry scores (PADUA, RENAL, and C-index) and conversion rates to open surgery. We also showed that the location of the tumor on the kidney might have an impact on conversion to open surgery. The upper pole and/or posterior localized tumor were found to be statistically higher in the CTO group in our study.

In the robotic area, the rate of conversion from MIS to open surgery was found to be lower for robotics than for laparoscopy. Prior studies have suggested that robotic surgery rather than laparoscopy is associated with less blood loss, shorter warm ischemia time, and shorter operative time; however, these parameters have unclear clinical significance for conversion to open surgery.^14^In our study, we did not find statistical differences between groups in preoperative ASA scores, the presence of comorbidities, or history of previous abdominal surgery. Furthermore, warm ischemia time was found to be similar between groups.

The regression analysis of our study showed that intraoperative blood loss and localization of tumors were significant predictive factors in both univariate and multivariate analyses for conversion to OPN. Posterior- and upper pole-located tumors was found to be significant preoperative predictors. The presence of posterior- and upper pole-located tumors was found to be a significant preoperative predictor. Although intraoperative blood loss was found to be a predictor of conversion to open surgery, it should not be forgotten that this value can only be effective peroperatively in the surgeon’s decision to convert to open surgery, since it cannot be predicted preoperatively.

Our study had several limitations. First, a relatively small number of patients were analyzed, and second, our study had a retrospective design and validation required a prospective study using multicenter data and larger sample sizes. Third, the rate of open conversion was also related to the surgeon’s experience and operation volume of the center. Our center is one of the biggest hospitals in our country. As a result, the surgeons who were included in this study are experienced and have had similar experience in laparoscopic surgery. However, including multiple surgeons in our study was a limitation.

## Conclusion

Open conversion should not be considered as a complication or failure in LMP. It is sometimes the safest option for patients when dealing with difficult tumors and nephron-sparing at the same time. Upper pole- and/or posterior-located tumors were found to be a preoperative predictor of conversion to open surgery. In addition, intraoperative blood loss was used as peroperative data to inform the surgeon about the decision and timing of conversion to open surgery. In clinical practice, when a high possibility of switching to open surgery is found using these factors, treatment planning will lead to the possibility of starting the treatment directly with open surgery instead of minimally invasive options, and it may also provide a chance to be prepared for the possibility of conversion to open surgery peroperatively.

## References

[1] Capitanio U, Bensalah K, Bex A, Boorjian SA, Bray F, Coleman J, Gore JL, Sun M, Wood C, Russo P (2019). Epidemiology of renal cell carcinoma. Eur Urol.

[2] Bukavina L, Bensalah K, Bray F, Carlo M, Challacombe B, Karam JA (2022). Epidemiology of renal cell carcinoma: 2022 update. Eur Urol.

[3] Guglielmetti GB, Dos Anjos GC, Sawczyn G, Rodrigues G, Cardili L, Cordeiro MD (2022). A prospective, randomized trial comparing the outcomes of open vs laparoscopic partial nephrectomy. J Urol.

[4] Huang WC, Elkin EB, Levey AS, Jang TL, Russo P (2009). Partial nephrectomy versus radical nephrectomy in patients with small renal tumors—Is there a difference in mortality and cardiovascular outcomes?. J Urol.

[5] Capitanio U, Terrone C, Antonelli A, Minervini A, Volpe A, Furlan M (2015). Nephron-sparing techniques independently decrease the risk of cardiovascular events relative to radical nephrectomy in patients with a T1a–T1b renal mass and normal preoperative renal function. Eur Urol.

[6] Kates M, Badalato GM, Pitman M, McKiernan JM (2011). Increased risk of overall and cardiovascular mortality after radical nephrectomy for renal cell carcinoma 2 cm or less. J Urol.

[7] Huang WC, Levey AS, Serio AM, Snyder M, Vickers AJ, Raj GV (2006). Chronic kidney disease after nephrectomy in patients with renal cortical tumours: a retrospective cohort study. Lancet Oncol.

[8] Wınfıeld HN, Donovan JF, Godet AS, Clayman RV (1993). Laparoscopic partial nephrectomy: initial case report for benign disease. J Endourol.

[9] Rais-Bahrami S, Lima GC, Varkarakis IM, Romero FR, Trock B, Jarrett TW (2006). Intraoperative conversion of laparoscopic partial nephrectomy. J Endourol.

[10] Khanna A, Campbell SC, Murthy PB, Ericson KJ, Nyame YA, Abouassaly R (2020). Unplanned conversion from minimally invasive to open kidney surgery: the impact of robotics. J Endourol.

[11] Kim FJ, Rha KH, Hernandez F, Jarrett TW, Pinto PA, Kavoussi LR (2003). Laparoscopic radical versus partial nephrectomy: assessment of complications. J Urol.

[12] Ramani AP, Desai MM, Steinberg AP, Ng CS, Abreu SC, Kaouk JH (2005). Complications of laparoscopic partial nephrectomy in 200 cases. J Urol.

[13] Yoshikawa Y, Ono Y, Hattori R, Gotoh M, Yoshino Y, Katsuno S (2004). Laparoscopic partial nephrectomy for renal tumor: Nagoya experience. Urology.

[14] Leow JJ, Heah NH, Chang SL, Chong YL, Png KS (2016). Outcomes of robotic versus laparoscopic partial nephrectomy: an updated meta-analysis of 4,919 patients. J Urol.

